# Oral Manifestation Like Forchheimer Spots of Dengue Fever

**DOI:** 10.4269/ajtmh.19-0338

**Published:** 2019-10

**Authors:** Kei Yamamoto

**Affiliations:** Disease Control and Prevention Center, National Center for Global Health and Medicine, Tokyo, Japan

**Figure 1.**
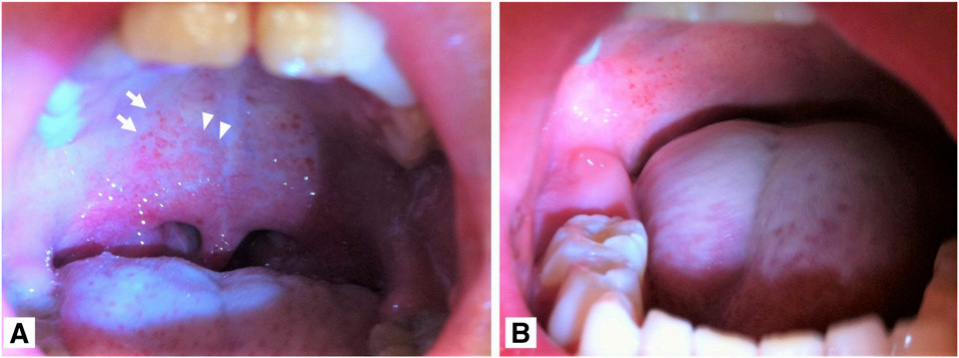
Oral manifestation. (**A**) Oral findings on day 4 of the illness showing spotted submucosal hemorrhages on the hard palate (arrow) and rose-colored spots on the soft palate (arrow head). (**B**) Oral findings on day 7 of the illness showing spotted submucosal hemorrhages on the hard palate only. This figure appears in color at www.ajtmh.org.

A 6-year-old boy of a mixed Japanese and Caucasian origin complained of a 4-day history of fever, cough, and blepharedema in 2014. He had a medical history of bronchial asthma and attention-deficit/hyperactivity disorder treated by methylphenidate. Blood test results showed a mild decrease in white blood cell (1,990/µL) and thrombocyte counts (9.6 × 10^4^/µL) without liver and kidney dysfunction. Dengue NS1 antigen was tested, and the results were positive. The serotype of dengue virus was type 1. The patient had no history of travel abroad in the last 2 weeks; however, an outbreak of dengue fever occurred in 2014 in Japan. He had spotted submucosal hemorrhages on the hard palate (Figure 1A, arrow) and rose-colored spots on the soft palate (Figure 1A, arrow head). Later, his fever receded, and a diffuse rash appeared on day 6 of the illness without severe complications. The rose-colored spots improved on day 7 of the illness (Figure 1B). Furthermore, he did not show any symptoms specific to rubella, measles, and scarlet fever, which occasionally involve the appearance of Forschheimer spots. Except for submucosal hemorrhages, oral manifestations of dengue infection were not common (less than 10%),^1,2^ but some authors reported as common findings (14–43%)^3,4^ as diffuse mucosal erythema or mucosal involvement. Moreover, Siler et al.^5^ reported that 19% of dengue fever patients produced experimentally by bites of infected mosquitoes had congestion of throat. However, the red spots on the soft palate such as Forchheimer spots^6^ have not been reported in the literature.
